# Triple-Negative Metaplastic Breast Carcinoma: Association of Epidermal Growth Factor Receptor Expression With Prognostic Parameters and Clinical Outcome

**DOI:** 10.7759/cureus.15006

**Published:** 2021-05-13

**Authors:** Shahzeb Munawar, Rimsha Haider, Syed Munqaad Ali, Syed Rafay Yaqeen, Sabeeh Islam, Ishaq Azeem Asghar, Anoshia Afzal, Shamail Zia, Muhammad Irfan, Atif A Hashmi

**Affiliations:** 1 Internal Medicine, Liaquat College of Medicine and Dentistry, Karachi, PAK; 2 Internal Medicine, Liaquat National Hospital and Medical College, Karachi, PAK; 3 Emergency Medicine, National Institute of Blood Diseases and Bone Marrow Transplantation, Karachi, PAK; 4 Internal Medicine, Dow University of Health Sciences, Karachi, PAK; 5 Internal Medicine, Baqai Medical University, Karachi, PAK; 6 Internal Medicine, St. Vincent Health Center, Buffalo, USA; 7 Internal Medicine, Faisalabad Medical University, Faisalabad, PAK; 8 Pathology, Ascension St. John Hospital, Detroit, USA; 9 Pathology, University of Oklahoma Health Sciences Center, Oklahoma City, USA; 10 Pathology, Ziauddin University, Karachi, PAK; 11 Statistics, Liaquat National Hospital and Medical College, Karachi, PAK; 12 Pathology, Liaquat National Hospital and Medical College, Karachi, PAK

**Keywords:** progesterone receptor, human epidermal growth factor receptor 2, metaplastic breast carcinoma, triple-negative breast carcinoma, epidermal growth factor receptor, estrogen receptor

## Abstract

Introduction

Metaplastic breast carcinoma (MBC) is one of the rare special subtypes of breast carcinoma associated with poor prognostic features compared with invasive ductal carcinoma. Moreover, therapeutic options are limited in MBC owing to frequent triple-negative profiles of these tumors. Epidermal growth factor receptor (EGFR) is a proto-oncogene that is overexpressed in many human cancers, and is a potential therapeutic target. Therefore, in this study, we evaluated the expression of EGFR in MBC by immunohistochemistry, and its association with clinicopathological and prognostic parameters.

Methods

We conducted a retrospective observational study in the Department of Histopathology at Liaquat National Hospital and Medical College, Pakistan, over a period of seven years. A total of 61 cases with a histopathological diagnosis of MBC were included in the study. All slides were reviewed by histopathologists for diagnostic confirmation. Histopathological parameters, such as tumor size, grade, and nodal metastasis, were recorded. The representative tissue blocks were also retrieved and immunohistochemical studies were performed for cytokeratin 5/6 (CK5/6), Ki67, and EGFR.

Results

The mean age of the patients was 44.48 ± 13.01 years. The mean tumor size was 5.72 ± 2.72 cm, with most of the cases belonging to tumor (T)-stage T3. Axillary metastasis was present in 57.4% cases, and the perinodal extension was present in 11.5% cases. Most tumors were grade III (85.2%), with a mean Ki67 index of 39.67% ± 20.38%. Most of the cases were nonbasal (83.6%), owing to the absent CK5/6 expression. Tumor recurrence was noted in 14.8% cases, with a median follow-up of 43 (13-83) months and median disease-free survival of 36 (12-60) months. Positive EGFR expression was noted in 52.5% cases. A significant association of EGFR expression was noted with tumor grade, mean Ki67 index, axillary metastasis, and nodal (N)-stage. Cases with positive EGFR expression were found to have higher grade (grade III), with higher Ki67 index, higher frequency of axillary metastasis, and higher N-stage. Moreover, cases with positive EGFR expression had lower disease-free survival compared to cases with negative EGFR expression.

Conclusion

We found that a significant proportion of triple-negative MBC expressed EGFR. Moreover, EGFR overexpression was associated with poor pathological parameters and lower disease-free survival. Therefore, EGFR can be considered a potential prognostic biomarker and therapeutic target in triple-negative MBC; however, the correlation between gene amplification and protein overexpression is required to better uncover the role of EGFR as a therapeutic target.

## Introduction

Metaplastic breast carcinoma (MBC) is one of the rare special subtypes of breast carcinoma associated with poor prognostic features compared with invasive breast ductal carcinoma [1,2]. These tumors tend to have a large tumor size at the time of presentation, and, therefore, require neoadjuvant chemotherapy [3]. However, the response of these tumors to neoadjuvant chemotherapy is poor, with a complete pathological response rate ranging from 10% to 17% [4]. Moreover, therapeutic options are limited owing to frequent triple-negative profiles of these tumors. Triple-negative breast tumors are defined by the lack of expression of estrogen receptor (ER), progesterone receptor (PR), and human epidermal growth factor receptor 2 (HER2/neu), and, therefore, they do not respond to hormonal and Herceptin therapy. Previous studies have reported a relatively high percentage of triple-negative breast tumors in Pakistan, and a significant percentage included MBC [5,6]. Epidermal growth factor receptor (EGFR) is a proto-oncogene that is overexpressed in many human cancers, and is a potential therapeutic target [7-9]. Although molecular studies are considered the gold standard to assess EGFR amplification, immunohistochemistry (IHC) to evaluate protein overexpression is considered surrogate to gene amplification studies. Therefore, in this study, we evaluated the expression of EGFR in MBC by IHC, and its association with clinicopathological and prognostic parameters.

## Materials and methods

We conducted a retrospective observational study in the Department of Histopathology at Liaquat National Hospital and Medical College, Pakistan, over a period of seven years. The specimens included were lumpectomy with or without axillary lymph node dissection, simple mastectomy, and modified radical mastectomy. Total 61 cases with a histopathological diagnosis of MBC were included in the study. Cases with primary breast cancer without evidence of systemic metastasis undergoing upfront tumor resection were included in the study. Cases with neoadjuvant chemoradiation were excluded from the study. Cases with clinically positive axillary lymph nodes underwent axillary lymph node dissection along with breast surgery. Those with clinically negative axillary lymph nodes were first subjected to sentinel lymph node (SLN) sampling with intraoperative frozen section analysis. Cases with positive SLNs on frozen section (>2 mm tumor size) were followed by axillary lymph node dissection. All slides were reviewed by histopathologists for diagnostic confirmation. Histopathological parameters, such as tumor size, grade, and nodal metastasis, were recorded.

**Figure 1:**
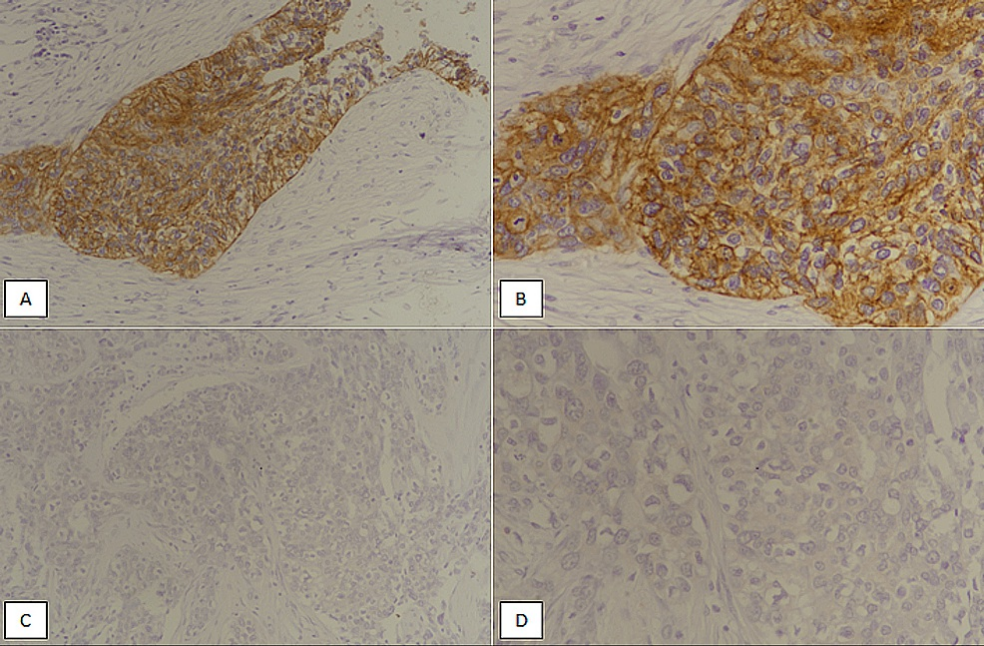
EGFR expression in triple-negative metaplastic breast carcinoma. (A) IHC staining at 200x magnification showing membranous expression of EGFR. (B) Positive EGFR expression at 400x magnification. (C): IHC staining at 200x magnification depicting negative EGFR expression. (D) Negative EGFR expression at 400x magnification. EGFR, epidermal growth factor receptor; IHC, immunohistochemical

The representative tissue blocks were also retrieved and immunohistochemical studies were performed for cytokeratin 5/6 (CK5/6), Ki67 and EGFR. The membranous EGFR expression was evaluated as described in previous studies [10,11]. Complete membranous expression in more than 10% invasive tumor cells was taken as positive EGFR expression (Figure 1A, 1B).

Ki67 was interpreted quantitatively and reported as the average percentage of positively stained tumor cells. Moderate-to-strong cytoplasmic CK5/6 expression in more than 10% invasive cancer cells was taken as positive CK5/6 expression. CK5/6 IHC was used to differentiate between the basal and nonbasal subtypes of triple-negative tumors. CK5/6-expressing tumors were labeled as basal subtype.

ER, PR, and HER2/neu IHC was also performed to confirm the triple-negative status, and the results were interpreted as described in previous studies [12-15]. More than 1% nuclear expression of ER and PR was taken as positive ER/PR expression. For HER2/neu, strong and complete membranous expression in more than 10% invasive cancer cells was taken as positive HER2/neu IHC. Cases with equivocal HER2/neu immunohistochemical results were confirmed by fluorescence in situ hybridization (FISH) testing. Cases with positive ER, PR, or HER2/neu IHC/FISH results were excluded from the study.

Data analysis was performed using Statistical Package for Social Sciences (Version 26.0, IBM Inc., Armonk, NY, USA). Chi-square, independent t-test, and Fisher’s exact tests were used to check the association. Survival analysis was done by the Kaplan-Meier method. P-values <0.05 were considered significant.

## Results

The mean age of the patients was 44.48 ± 13.01 years. The mean tumor size was 5.72 ± 2.72 cm, with most of the cases belonging to tumor (T)-stage T3. Axillary metastasis was present in 57.4% cases, and the perinodal extension was present in 11.5% cases. Most of the tumors were grade III (85.2%), with a mean Ki67 index of 39.67% ± 20.38%. Most of the cases were nonbasal (83.6%), owing to the absent CK5/6 expression. Tumor recurrence was noted in 14.8% cases, with a median follow-up of 43 (13-83) months and median disease-free survival of 36 (12-60) months. Positive EGFR expression was noted in 52.5% cases (Table 1).

**Table 1:** Clinicopathological features of population under study SD, standard deviation; N, nodal; T, tumor; EGFR, epidermal growth factor receptor

Clinicopathological features	Values
Age (years); mean ± SD	44.48 ± 13.01
Age groups	
≤50 years, n (%)	29 (47.5)
>50 years, n (%)	32 (52.5)
Tumor size (cm); mean ± SD	5.72 ± 2.72
Ki67 index (%); mean ± SD	39.67 ± 20.38
Ki67 index groups	
≤24%, n (%)	20 (32.8)
25%-44%, n (%)	16 (26.2)
>44%, n (%)	25 (41)
Disease-free survival (months); median (range)	36 (12–60)
Axillary metastasis	
Present, n (%)	35 (57.4)
Absent, n (%)	26 (42.6)
N-stage	
N0, n (%)	26 (42.6)
N1, n (%)	16 (26.2)
N2, n (%)	9 (14.8)
N3, n (%)	10 (16.4)
Perinodal extension	
Present, n (%)	7 (11.5)
Absent, n (%)	54 (88.5)
T-stage	
T1, n (%)	5 (8.2)
T2, n (%)	18 (29.5)
T3, n (%)	38 (62.3)
Tumor grade	
Grade II, n (%)	9 (14.8)
Grade III, n (%)	52 (85.2)
Surgery type	
Modified radical mastectomy, n (%)	50 (82)
Simple mastectomy, n (%)	11 (18)
Necrosis	
Absent, n (%)	13 (12.3)
Focal, n (%)	22 (36.1)
Marked, n (%)	26 (42.6)
Fibrosis	
Mild, n (%)	7 (11.5)
Moderate, n (%)	34 (55.7)
Severe, n (%)	20 (32.8)
Lymphocytic infiltration	
Absent, n (%)	5 (8.2)
Moderate, n (%)	39 (63.9)
Severe, n (%)	17 (27.9)
In situ component	
Present, n (%)	21 (34.4)
Absent, n (%)	40 (65.6)
Lymphovascular invasion	
Present, n (%)	27 (44.3)
Absent, n (%)	34 (55.7)
Triple-negative subtype	
Basal, n (%)	10 (16.4)
Nonbasal, n (%)	51 (83.6)
Recurrence	
Yes, n (%)	9 (14.8)
No, n (%)	52 (85.2)
EGFR	
Positive, n (%)	32 (52.5)
Negative, n (%)	29 (47.5)

Table 2 depicts the association of EGFR expression with clinicopathological features. A significant association of EGFR expression was noted with tumor grade, mean Ki67 index, axillary metastasis, and nodal (N)-stage. Cases with positive EGFR expression were found to have higher grade (grade III), with higher Ki67 index, higher frequency of axillary metastasis, and higher N-stage.

**Table 2:** Association of clinicopathological features with EGFR expression *Chi-square test was applied, **Fisher’s exact test was applied, ***independent t-test was applied, ****significant at <0.05 EGFR, epidermal growth factor receptor; SD, standard deviation; N, nodal; T, tumor

Clinicopathological features	Values		P-value
	EGFR expression		
	Positive	Negative	
Age (years); mean ± SD***	44.72 ± 13.22	44.21 ± 13.01	0.880
Age group*			
≤50 years, n (%)	27 (84.4)	13 (44.8)	0.001****
>50 years, n (%)	5 (15.6)	16 (55.2)	
Tumor size (cm); mean ± SD***	5.38 ± 2.31	6.10 ± 3.10	0.300
Ki67 index (%); mean ± SD***	44.53 ± 24.30	34.31 ± 13.41	0.045****
Axillary metastasis*			
Present, n (%)	27 (84.4)	8 (27.6)	<0.0001****
Absent, n (%)	5 (15.6)	21 (72.4)	
N-stage**			
N0, n (%)	5 (15.6)	21 (71.4)	<0.0001****
N1, n (%)	8 (25)	8 (27.6)	
N2, n (%)	9 (28.1)	0 (0)	
N3, n (%)	10 (31.3)	0 (0)	
T-stage**			
T1, n (%)	5 (15.6)	0 (0)	0.060
T2, n (%)	7 (21.9)	11 (37.9)	
T3, n (%)	20 (62.5)	18 (62.1)	
Tumor grade**			
Grade II, n (%)	1 (3.1)	8 (27.6)	0.010****
Grade III, n (%)	31 (96.9)	21 (72.4)	
Triple-negative subtype**			
Basal, n (%)	7 (21.9)	3 (10.3)	0.307
Nonbasal, n (%)	25 (78.1)	26 (89.7)	
Recurrence**			
Yes, n (%)	7 (21.9)	2 (6.9)	0.151
No, n (%)	25 (78.1)	27 (93.1)	

**Figure 2:**
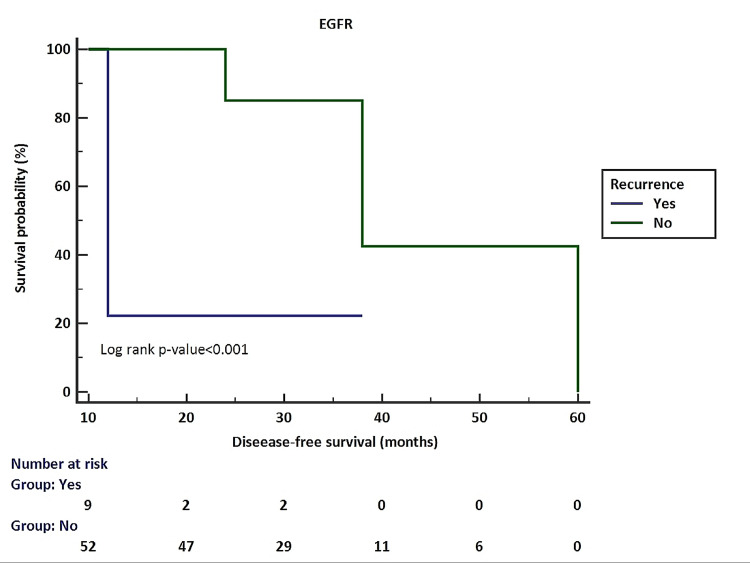
Association of EGFR expression with disease-free survival EGFR, epidermal growth factor receptor

Figure 2 shows the association of EGFR expression with disease-free survival. Cases with positive EGFR expression had lower disease-free survival compared to cases with negative EGFR expression.

## Discussion

In this study, we evaluated the prognostic significance of EGFR expression in triple-negative MBCs. We found that a significant proportion of cases of triple-negative MBC had positive EGFR expression. Moreover, EGFR expression was associated with poor prognostic parameters, such as higher tumor grade, higher mean Ki67 index, poor disease-free survival, and higher frequency of axillary metastasis.

MBCs have been reported to carry EGFR overexpression in up to 80% of cases, and approximately one-third of those cases carry EGFR gene amplification [16]. Reddy et al. reviewed triple-negative MBCs in relation to the previously linked epithelial-to-mesenchymal molecular alterations, and concluded that MBCs were aggressive tumors with poor prognostic features and overall outcome, especially in the presence of EGFR amplification [17].

Downs-Kelly et al. reported similar findings with increased local and distant recurrence with MBCs [18]. Song et al. subclassified MBCs and reported a bad outcome for most MBCs compared with other triple-negative breast cancers. They further endorsed other studies by reporting a larger tumor size, a higher percentage of ER/PR negativity, and a higher Ki67 index in MBC [19]. Similarly, Gilbert et al. reported high copy numbers of EGFR in MBC due to aneusomy and amplification and recommended further trials with targeted therapies [20].

McCart Reed et al. studied phenotypic and molecular features of MBC and reported EGFR overexpression as one of the most important negative prognostic factors [21]. However, some studies have reported a lack of relationship between EGFR overexpression and the actual presence of EGFR mutation [22,23], and it is, therefore, recommended to correlate these cases with molecular studies to select a target population for a better treatment response with EGFR inhibitors [22-24]. Gumuskaya et al. reported a positive association of membranous staining pattern of EGFR expression with EGFR gene amplification (increased gene copy number) compared to the cytoplasmic staining pattern and recommended to prioritize those patients for anti-EGFR treatment [25].

Our study had a few limitations, as it represents single-institution data. Moreover, molecular studies were not performed to assess EGFR amplification. Therefore, we advice that large-scale studies with molecular correlation should be conducted in our population to determine the prognostic significance of EGFR as a biomarker in triple-negative MBC.

## Conclusions

We found that EGFR expression in triple-negative MBC signifies poor prognostic significance, as positive EGFR expression was significantly associated with axillary metastasis, higher tumor grade, and higher mean Ki67 index. Moreover, EGFR-positive cases had poor disease-free survival when compared to cases with negative EGFR expression. We noted that a significant proportion of triple-negative MBC had EGFR expression; therefore, EGFR can serve as a potential therapeutic target in triple-negative MBC. However, further studies are needed to find the correlation of gene amplification with protein expression to better evaluate the therapeutic response.

## References

[1] Haroon S, Zia S, Shirazi U (2021). Metaplastic breast carcinoma: clinicopathological parameters and prognostic profile. Cureus.

[2] Hashmi AA, Aijaz S, Mahboob R (2018). Clinicopathologic features of invasive metaplastic and micropapillary breast carcinoma: comparison with invasive ductal carcinoma of breast. BMC Res Notes.

[3] Ong CT, Campbell BM, Thomas SM (2018). Metaplastic breast cancer treatment and outcomes in 2500 patients: a retrospective analysis of a national oncology database. Ann Surg Oncol.

[4] Al-Hilli Z, Choong G, Keeney MG, Visscher DW, Ingle JN, Goetz MP, Jakub JW (2019). Metaplastic breast cancer has a poor response to neoadjuvant systemic therapy. Breast Cancer Res Treat.

[5] Hashmi AA, Naz S, Hashmi SK (2018). Prognostic significance of p16 & p53 immunohistochemical expression in triple negative breast cancer. BMC Clin Pathol.

[6] Hashmi AA, Edhi MM, Naqvi H, Faridi N, Khurshid A, Khan M (2014). Clinicopathologic features of triple negative breast cancers: an experience from Pakistan. Diagn Pathol.

[7] Hashmi AA, Naz S, Hashmi SK (2019). Epidermal growth factor receptor (EGFR) overexpression in triple-negative breast cancer: association with clinicopathologic features and prognostic parameters. Surg Exp Pathol.

[8] Hashmi AA, Hashmi SK, Irfan M (2019). Prognostic utility of epidermal growth factor receptor (EGFR) expression in prostatic acinar adenocarcinoma. Appl Cancer Res.

[9] Hashmi AA, Hussain ZF, Irfan M (2019). Epidermal growth factor receptor (EGFR) overexpression in endometrial carcinoma: association with histopathologic parameters. Surg Exp Pathol.

[10] Hashmi AA, Hussain ZF, Aijaz S (2018). Immunohistochemical expression of epidermal growth factor receptor (EGFR) in South Asian head and neck squamous cell carcinoma: association with various risk factors and clinico-pathologic and prognostic parameters. World J Surg Oncol.

[11] Hashmi AA, Hussain ZF, Irfan M (2018). Prognostic significance of epidermal growth factor receptor (EGFR) over expression in urothelial carcinoma of urinary bladder. BMC Urol.

[12] Hashmi AA, Munawar S, Rehman N (2021). Invasive papillary carcinoma of the breast: clinicopathological features and hormone receptor profile. Cureus.

[13] Hashmi AA, Zia S, Yaqeen SR (2021). Mucinous breast carcinoma: clinicopathological comparison with invasive ductal carcinoma. Cureus.

[14] Hashmi AA, Iftikhar SN, Munawar S, Shah A, Irfan M, Ali J (2020). Encapsulated papillary carcinoma of breast: clinicopathological features and prognostic parameters. Cureus.

[15] Hashmi AA, Iftikhar SN, Haider R, Haider R, Irfan M, Ali J (2020). Solid papillary carcinoma of breast: clinicopathologic comparison with conventional ductal carcinoma of breast. Cureus.

[16] Reis-Filho JS, Pinheiro C, Lambros MB (2006). EGFR amplification and lack of activating mutations in metaplastic breast carcinomas. J Pathol.

[17] Reddy TP, Rosato RR, Li X, Moulder S, Piwnica-Worms H, Chang JC (2020). A comprehensive overview of metaplastic breast cancer: clinical features and molecular aberrations. Breast Cancer Res.

[18] Downs-Kelly E, Nayeemuddin KM, Albarracin C, Wu Y, Hunt KK, Gilcrease MZ (2009). Matrix-producing carcinoma of the breast: an aggressive subtype of metaplastic carcinoma. Am J Surg Pathol.

[19] Song Y, Liu X, Zhang G (2013). Unique clinicopathological features of metaplastic breast carcinoma compared with invasive ductal carcinoma and poor prognostic indicators. World J Surg Oncol.

[20] Gilbert JA, Goetz MP, Reynolds CA (2008). Molecular analysis of metaplastic breast carcinoma: high EGFR copy number via aneusomy. Mol Cancer Ther.

[21] McCart Reed AE, Kalaw E, Nones K (2019). Phenotypic and molecular dissection of metaplastic breast cancer and the prognostic implications. J Pathol.

[22] Teng YH, Tan WJ, Thike AA (2011). Mutations in the epidermal growth factor receptor (EGFR) gene in triple negative breast cancer: possible implications for targeted therapy. Breast Cancer Res.

[23] Willmore-Payne C, Holden JA, Layfield LJ (2006). Detection of epidermal growth factor receptor and human epidermal growth factor receptor 2 activating mutations in lung adenocarcinoma by high-resolution melting amplicon analysis: correlation with gene copy number, protein expression, and hormone receptor expression. Hum Pathol.

[24] Masuda H, Zhang D, Bartholomeusz C, Doihara H, Hortobagyi GN, Ueno NT (2012). Role of epidermal growth factor receptor in breast cancer. Breast Cancer Res Treat.

[25] Gumuskaya B, Alper M, Hucumenoglu S, Altundag K, Uner A, Guler G (2010). EGFR expression and gene copy number in triple-negative breast carcinoma. Cancer Genet Cytogenet.

